# Epidemiology and economic burden of Von Hippel-Lindau Disease-associated central nervous system hemangioblastomas and pancreatic neuroendocrine tumors in the United States

**DOI:** 10.1186/s13023-024-03060-w

**Published:** 2024-02-16

**Authors:** Eric Jonasch, Yan Song, Jonathan Freimark, Richard Berman, Ha Nguyen, James Signorovitch, Murali Sundaram

**Affiliations:** 1 The University of Texas MD Anderson Cancer Center, 1515 Holcombe Blvd, 77030 Houston, TX USA; 2 Analysis Group, Inc., 111 Huntington Ave, 02199 Boston, MA USA; 3 Merck & Co., Inc., 126 E. Lincoln Ave., 07065 Rahway, NJ USA

**Keywords:** Central nervous system hemangioblastoma, Economic burden, Epidemiology, Pancreatic neuroendocrine tumor, Von Hippel-Lindau disease

## Abstract

**Background:**

To date, real-world evidence around the clinical and economic burden related to von Hippel-Lindau (VHL) disease is limited. Therefore, this study characterized the prevalence, healthcare resource utilization (HRU), and economic burden of von Hippel-Lindau–associated central nervous system hemangioblastoma (VHL-CNS-Hb) and pancreatic neuroendocrine tumors (VHL-pNET) in the United States (US).

**Methods:**

Patients with VHL-CNS-Hb or VHL-pNET were identified from Optum’s de-identified Clinformatics® Data Mart Database (2007–2020) and matched 1:5 to control patients without VHL disease or CNS-Hb/pNET. Prevalence rates of VHL-CNS-Hb and VHL-pNET (standardized by age and sex) in 2019 were estimated. HRU and healthcare costs (2020 US dollars) were compared between the VHL-CNS-Hb/VHL-pNET and control cohorts.

**Results:**

In 2019, US prevalence rates of VHL-CNS-Hb and VHL-pNET were estimated to be 1.12 cases per 100,000 (3,678 patients) and 0.12 cases per 100,000 (389 patients), respectively. Patients with VHL-CNS-Hb (*N* = 220) had more inpatient, outpatient, and emergency department visits and $49,645 higher annual healthcare costs than controls (*N* = 1,100). Patients with VHL-pNET (*N* = 20) had more inpatient and outpatient visits and $56,580 higher annual healthcare costs than controls (*N* = 100). Costs associated with surgical removal of CNS-Hb and pNET were particularly high.

**Conclusions:**

In this retrospective, claims-based study, both VHL-CNS-Hb and VHL-pNET were associated with substantial HRU and healthcare costs, particularly tumor reduction surgery–related costs. These findings provide important insight for healthcare payers regarding the expected real-world costs that enrollees with VHL-CNS-Hb and VHL-pNET may incur over the course of their disease.

**Supplementary Information:**

The online version contains supplementary material available at 10.1186/s13023-024-03060-w.

## Introduction

Von Hippel-Lindau (VHL) disease is an inherited tumor syndrome characterized by the development of multiple benign and malignant tumors and cysts in various internal organs, including the nervous system [1, 2]. The epidemiology of VHL disease varies by country and population, with some European studies estimating prevalence rates of 1 in 91,111 to 1 in 38,951 [3–6]. Meanwhile, in the United States (US), a recent study estimated a VHL disease prevalence of 2.13 per 100,000 in 2019 based on health insurance claims data [7]. 

While patients with VHL disease may develop various types of tumors, including clear cell renal cell carcinomas (RCC), pancreatic neuroendocrine tumors (pNETs), pheochromocytomas and paragangliomas, and endolymphatic sac tumors, central nervous system hemangioblastoma (CNS-Hb) is typically the first manifestation of VHL disease [2], occurring in 70-80% of cases [8]. VHL-associated CNS-Hb (VHL-CNS-Hb) is generally slow-growing and multifocal, with symptoms varying depending on location of the tumor (e.g., gait ataxia for cerebellar CNS-Hb and hypesthesia for spinal CNS-Hb) [2, 8]. In contrast, VHL-associated pNETs (VHL-pNET), which occur in 9-17% of patients with VHL disease, tend to be asymptomatic; however, when symptoms do appear, they may include abdominal pain, jaundice, and pancreatitis [1]. 

Management of VHL-CNS-Hb and VHL-pNET mainly centers around surgery [2]. Asymptomatic VHL-CNS-Hb may be managed with active surveillance, but surgical resection is recommended for symptomatic tumors or those causing cerebrospinal fluid obstruction [2, 9]. For VHL-pNET, tumors with a diameter larger than 2–3 cm should be surgically removed to avoid metastasis [1, 2, 10]. However, the need for multiple surgeries for multifocal VHL tumors can lead to deterioration of performance status and increased risk of surgical complications, emphasizing the unmet treatment needs of patients with VHL-associated tumors [8, 10]. 

Notably, treatment options for VHL disease have recently broadened with the introduction of belzutifan, a hypoxia-inducible factor 2α (HIF-2α) inhibitor approved by the Food and Drug Administration (FDA) in August 2021 for the treatment of VHL-associated RCC, CNS-Hb, and pNET that do not require immediate surgery [11, 12]. In the pivotal phase 2 clinical trial, an objective response was seen with belzutifan treatment in 30% of patients with VHL-CNS-Hb and 77% of patients with VHL-pNET [11]. 

To date, real-world evidence around the clinical and economic burden related to VHL disease is limited. Accordingly, a retrospective study was recently conducted to develop an algorithm to identify patients with VHL disease from insurance claims databases and to characterize the healthcare resource utilization (HRU) and economic burden associated with VHL-RCC in real-world clinical practice [7]. The current study leverages this algorithm to examine two additional main tumor manifestations of VHL disease — CNS-Hb and pNET, thereby filling the literature gap related to the HRU and healthcare costs associated with VHL disease.

## Methods

### Data source

Claims data from 2007 to 2020 were used from Optum’s de-identified Clinformatics® Data Mart Database (CDM). The CDM dataset is a single-payer source of US claims data for more than 15 million lives in any given year. The population is predominantly composed of patients from the South and North Central (Midwest) census regions, though there are members from all 50 states. All patients aged 65 + are in commercial plans or Medicare Advantage plans. Data were de-identified and comply with the patient requirements of the Health Insurance Portability and Accountability Act (HIPAA) of 1996; therefore, no reviews by an institutional review board were required. Because of Optum’s privacy policies associated with the CDM dataset, when variable categories are presented in results tables and there are patient frequency counts < 5, these results are shown as “<5”.

### Study population and identification of patients with VHL disease

**Fig. 1:**
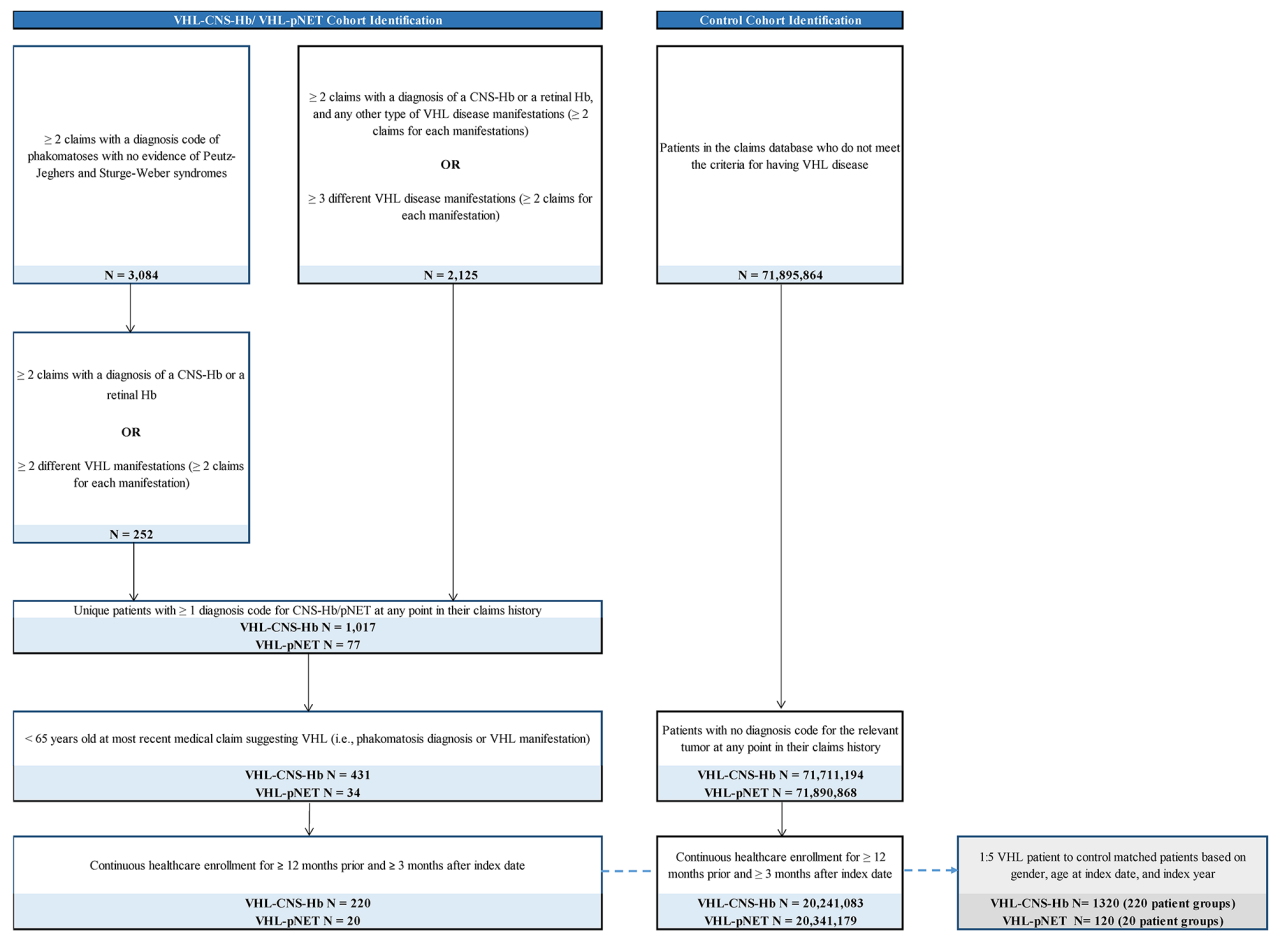
Sample selection of patient cohorts^1^. Abbreviations: CNS: central nervous system; Hb: hemangioblastoma; ICD: International Classification of Diseases; pNET: pancreatic neuroendocrine tumor; VHL: von Hippel-Lindau. Note: 1. See Jonasch et al. for the list of codes used in this VHL disease identification algorithm [7]

With the lack of an International Classification of Diseases (ICD) diagnosis code for VHL disease, a claims-based algorithm to identify patients with VHL disease was previously developed and validated (Fig. 1) [7]. Based on the algorithm, patients with a phakomatosis diagnosis were identified as having VHL disease if they had ≥ 2 claims with a diagnosis code for a group of phakomatoses that includes VHL disease (ICD-9 759.6 or ICD-10 Q85.8), and no evidence of cutaneous manifestations, gastrointestinal manifestations, or glaucoma, which are associated with Peutz-Jeghers and Sturge-Weber syndromes and are also captured by the diagnosis codes for phakomatoses. These patients were also required to have ≥ 2 claims with diagnoses for CNS hemangioblastoma or retinal hemangioblastoma (disease manifestations highly specific to VHL disease), or ≥ 2 different VHL disease manifestations with ≥ 2 claims including relevant diagnosis codes for each of the manifestations (including benign retinal neoplasms, pheochromocytoma, endolymphatic sac tumor, RCC, renal cysts, pancreatic tumors, pancreatic cysts, and papillary cystadenomas of the epididymis).

Patients without a phakomatosis diagnosis were identified as having VHL disease if they had ≥ 2 claims with diagnoses for CNS-Hb or retinal hemangioblastoma, and at least one other type of VHL disease manifestation with ≥ 2 claims including relevant diagnosis codes for that manifestation. Alternatively, patients without a phakomatosis diagnosis could have ≥ 3 different VHL disease manifestations with ≥ 2 claims including relevant diagnosis codes for each manifestation.

Among these patients with VHL disease, those with VHL-CNS-Hb or VHL-pNET were then identified as having ≥ 1 diagnosis code for CNS-Hb or pNET at any point in their claims history. Patients with VHL-CNS-Hb or VHL-pNET were included in the study if they were < 65 years of age at the most recent medical claim suggesting VHL disease (i.e., phakomatosis diagnosis or VHL disease manifestation), which was a criterion meant to exclude patients with sporadic tumors that were not associated with VHL, and had continuous healthcare enrollment for ≥ 12 months prior to and ≥ 3 months following the index date, which was defined as the date of the first observed CNS-Hb or pNET diagnosis.

Patients were included in the control cohort if they did not meet the aforementioned criteria for having VHL disease, had no diagnosis code for CNS-Hb or pNET at any point in their claims history, and had continuous healthcare enrollment for ≥ 12 months prior to and ≥ 3 months following the index date, which was the date of a random medical claim within the patient’s claims history. Patients in the VHL-CNS-Hb cohort and VHL-pNET cohort were matched 1:5 to control patients based on age at index date, sex, and index year.

### Study design

This study used a retrospective cohort design. The baseline period comprised the 12 months prior to the index date, while the study period spanned from the index date to the end of continuous healthcare coverage or data availability, or up to 5 years, whichever occurred first.

### Study outcomes

Outcomes measured in this study included the prevalence of VHL-CNS-Hb and VHL-pNET in 2019; all-cause HRU and healthcare costs; CNS-Hb and pNET-related HRU and medical costs; healthcare costs associated with VHL-related surgery (i.e., nephrectomy, laser therapy to the retina, and surgical removal of CNS-Hb, adrenal pheochromocytomas, and pNET); and rates and costs of CNS-Hb–related tumor reduction procedure complications. Since the sample size and number of complications observed in the VHL-pNET cohort were low, only the rates of pNET–related tumor reduction procedure complications were reported for this cohort.

All-cause and CNS-Hb and pNET-related HRU comprised the use of inpatient, outpatient, emergency department, and other medical services, reported as events per person-year. CNS-Hb- and pNET-related HRU was identified from claims for medical services that included a diagnosis code for CNS-Hb or pNET, respectively. Healthcare costs comprised medical (i.e., inpatient, outpatient, emergency department [ED], other medical services) and pharmacy components, reported as mean annual costs adjusted for inflation in 2020 US dollars (USD).

Complications associated with CNS-Hb and pNET reduction procedures were classified as any associated diagnoses or procedures related to a medical complication following the corresponding tumor reduction procedure. Long-term complications of these procedures, such as chronic pain and cerebral vasculature occlusion/stroke, were evaluated for up to 6 months after surgery. Short-term complications were considered up to 4 weeks from surgery and included events such as acute renal failure, hemorrhage, and thromboembolism.

### Statistical analysis

To calculate the prevalence of VHL disease, VHL-CNS-Hb, and VHL-pNET, the number of patients with each condition identified in the claims data with healthcare enrollment in 2019 and ≥ 12 months of continuous eligibility prior to their last healthcare enrollment date in 2019 was divided by the total number of patients in the database with healthcare enrollment in 2019 and ≥ 12 months of continuous eligibility prior to their last healthcare enrollment date in 2019. Prevalence was standardized using the age-sex distribution of the 2019 US population [13] and reported per 100,000 individuals.

Baseline characteristics were compared between the VHL-CNS-Hb or VHL-pNET cohorts and their respective matched control cohorts using univariable generalized estimating equation (GEE) models. Univariable generalized linear models with a negative binomial distribution were used to compare unadjusted all-cause HRU between the study cohorts, with incidence rate ratios (IRRs) and their corresponding 95% confidence intervals (CIs) and P-values reported. Univariable generalized linear models with a Tweedie distribution were used to compare all-cause healthcare costs, with incremental cost differences and P-values reported. Additionally, HRU and cost regression models were conducted adjusting for age at index date, sex, geographic region, health insurance type, and number of outpatient visits during the baseline period.

Costs of tumor reduction procedure complications were estimated via univariable generalized linear models, using a Tweedie distribution; these models compared costs between patients who had a complication versus those who did not, using the difference between the cohorts as the cost of the complication. Annual CNS-Hb- and pNET-related HRU incidence rates and medical costs, as well as average costs for VHL-related surgeries, were presented as unadjusted estimates for patients with VHL-CNS-Hb or VHL-pNET.

## Results

### Prevalence of VHL-CNS-Hb and VHL-pNET in 2019

In 2019, the prevalence of VHL-CNS-Hb was estimated to be 1.12 cases per 100,000, representing approximately 3,678 patients with VHL-CNS-Hb in the US (Supplemental Figure S1). The prevalence of VHL-pNET was estimated to be 0.12 cases per 100,000, representing approximately 389 patients with VHL-pNET in the US (Supplemental Figure S1).

### Baseline characteristics of VHL-CNS-Hb and VHL-pNET patients and controls

Among 220 VHL-CNS-Hb patients matched to 1,100 control patients, 61.8% and 61.6%, respectively, were female, and the average age in both cohorts was 49.0 years (Table 1). Mean Charlson Comorbidity Index (CCI) was significantly higher in the VHL-CNS-Hb cohort compared to the control cohort (2.0 vs. 0.4; *P* < 0.001), with numerous comorbidities occurring more commonly in the former. Additionally, annual baseline HRU and healthcare costs were significantly higher in the VHL-CNS-Hb cohort compared to the control cohort.

**Table 1:** Baseline Characteristics of VHL-CNS-Hb/VHL-pNET and Control Cohorts

	VHL-CNS-Hb		vs.	Control			VHL-pNET		vs.	Control		
	(*N* = 220)			(*N* = 1,100)			(*N* = 20)			(*N* = 100)		
**Demographic characteristics**												
Female, N (%)	136	(61.8%)		678	(61.6%)		12	(60.0%)		60	(60.0%)	
Age at index date (years), mean ± SD	49.0	± 11.3		49.0	± 11.2		47.1	± 14.1		47.1	± 13.8	
Geographic region, N (%)												
Northeast	22	(10.0%)		107	(9.7%)		< 5	-		8	(8.0%)	
Midwest	51	(23.2%)		284	(25.8%)		5	(25.0%)		19	(19.0%)	
South	108	(49.1%)		479	(43.5%)		10	(50.0%)		51	(51.0%)	
West	38	(17.3%)		220	(20.0%)		< 5	-		22	(22.0%)	
Unknown	< 5	-		10	(0.9%)		< 5	-		< 5	-	
Health insurance type, N (%)												
EPO	19	(8.6%)		108	(9.8%)		< 5	-		8	(8.0%)	
PPO	17	(7.7%)		42	(3.8%)		< 5	-		5	(5.0%)	
POS	123	(55.9%)		723	(65.7%)		12	(60.0%)		74	(74.0%)	
HMO	44	(20.0%)		184	(16.7%)		< 5	-		10	(10.0%)	
Other^1^	19	(8.6%)		42	(3.8%)		< 5	-		< 5	-	
**Disease characteristics**												
Year of index date, N (%)												
2018 − 2012	85	(38.6%)		425	(38.6%)		8	(40.0%)		40	(40.0%)	
2013–2016	75	(34.1%)		375	(34.1%)		< 5	-		10	(10.0%)	
2017–2020	60	(27.3%)		300	(27.3%)		10	(50.0%)		50	(50.0%)	
**Comorbidities**												
CCI, mean ± SD	2.0	± 2.6		0.4	± 1.0	***	2.8	± 3.6		0.3	± 0.8	***
Comorbidities, N (%)												
Hypertension	100	(45.5%)		327	(29.7%)	***	11	(55.0%)		28	(28.0%)	*
Depression/anxiety	73	(33.2%)		181	(16.5%)	***	9	(45.0%)		19	(19.0%)	*
Chronic pulmonary disease	54	(24.5%)		103	(9.4%)	***	< 5	-		12	(12.0%)	
Diabetes	34	(15.5%)		108	(9.8%)	*	7	(35.0%)		5	(5.0%)	**
Osteoporosis/fractures	30	(13.6%)		59	(5.4%)	***	< 5	-		6	(6.0%)	
Liver disease	37	(16.8%)		33	(3.0%)	***	< 5	-		< 5	-	*
Cerebrovascular disease	51	(23.2%)		27	(2.5%)	***	< 5	-		< 5	-	
Congestive heart failure	8	(3.6%)		23	(2.1%)		< 5	-		< 5	-	
Peripheral vascular disease	19	(8.6%)		22	(2.0%)	***	< 5	-		< 5	-	
Renal disease	24	(10.9%)		20	(1.8%)	***	< 5	-		< 5	-	
Cancer	50	(22.7%)		43	(3.9%)	***	7	(35.0%)		< 5	-	**
**Annual HRU during baseline, mean ± SD**												
Inpatient visits	0.7	± 1.7		0.1	± 0.6	***	1.5	± 3.7		0.1	± 0.5	
Inpatient days	2.9	± 7.4		0.8	± 7.5	***	4.0	± 5.9		0.3	± 1.0	*
ED visits	1.8	± 4.7		0.6	± 1.8	***	0.5	± 0.8		1.0	± 3.7	
Outpatient visits	15.2	± 10.6		7.7	± 8.8	***	20.1	± 11.3		7.1	± 8.3	***
Other visits^2^	1.8	± 2.9		0.8	± 2.2	***	2.1	± 3.2		0.5	± 1.3	*
**Annual healthcare costs during baseline, mean ± SD**												
Total costs	$44,688	± $92,113		$9,102	± $40,331	***	$51,768	± $76,003		$5,911	± $13,839	**
Medical costs	$38,869	± $87,536		$7,371	± $39,206	***	$46,657	± $75,751		$3,309	± $6,529	**
Inpatient costs	$21,270	± $75,640		$3,435	± $34,478	***	$9,443	± $13,806		$916	± $4,409	**
ED costs	$3,600	± $12,781		$635	± $2,579	***	$1,244	± $2,191		$443	± $1,458	
Outpatient costs	$13,519	± $36,804		$3,122	± $11,500	***	$35,221	± $76,519		$1,917	± $3,364	*
Other costs^2^	$480	± $2,513		$178	± $1,685	*	$749	± $1,514		$33	± $106	*
Pharmacy costs	$5,819	± $22,709		$1,731	± $7,090	**	$5,112	± $9,488		$2,602	± $11,823	

**Abbreviations**: CCI: Charlson Comorbidity Index; CNS: central nervous system; ED: emergency department; EPO: exclusive provider organization; GEE: generalized estimating equation; Hb: hemangioblastoma; HMO: health maintenance organization; HRU: healthcare resource utilization; ICD: International Classification of Diseases; pNET: pancreatic neuroendocrine tumor; POS: point of service; PPO: preferred provider organization; SD: standard deviation; USD: United States dollars; VHL: von Hippel-Lindau.

**Notes**:

* denotes P-values < 0.05; ** denotes P-values < 0.01; *** denotes P-values < 0.001

1. The “other” health insurance category includes the following types of plans: independent practice organizations, lock-ins, pharmacy network plans, unknown, and no coverage

2. “Other” medical visits/costs include all those not captured by inpatient, ED, or outpatient visits/costs. These claims may reflect home health aids or physician visits among other types of ancillary medical claims

3. The dashed cells (“-“) are used for variable categories where there are < 5 patients. According to Optum’s data rules, these numbers need to be redacted to ensure patient privacy

Among 20 VHL-pNET patients matched to 100 control patients, 60.0% were female and the average age was 47.1 years in both cohorts (Table 1). Similar to VHL-CNS-Hb patients and their controls, differences in CCI (2.8 vs. 0.3; *P* < 0.001), HRU, and healthcare costs were observed.

### HRU and healthcare costs

During the study period, the VHL-CNS-Hb cohort incurred a higher mean number of inpatient admissions (1.1 vs. 0.2; adjusted IRR [95% CI] = 4.7 [3.3, 6.7]; *P* < 0.001), outpatient visits (17.8 vs. 8.9; adjusted IRR [95% CI] = 1.6 [1.4, 1.8]; *P* < 0.001), ED visits (1.7 vs. 0.7; adjusted IRR [95% CI] = 2.0 [1.3, 3.0]; *P* < 0.01), and other medical visits (3.2 vs. 1.2; adjusted IRR [95% CI] = 2.7 [1.8, 4.2]; *P* < 0.001) per person-year, as well as longer mean inpatient length of stay (9.3 vs. 0.9 days per person-year; adjusted IRR [95% CI] = 11.5 [6.3, 21.2]; *P* < 0.001), relative to the control cohort (Table 2). Accordingly, higher total annual healthcare costs were observed in the VHL-CNS-Hb cohort compared to the control cohort ($70,924 vs. $11,418; adjusted cost difference = $49,645; *P* < 0.001), with medical costs representing the largest component ($61,919 vs. $9,094; adjusted cost difference = $45,090; *P* < 0.001; Table 3).

**Table 2:** HRU in Patients with VHL-CNS-Hb or VHL-pNET

	Tumor-Related Incidence Rates (events/100 person-years)^2,3^	All-Cause Adjusted Incidence Rates (events/person-year)		All-Cause HRU IRR			
Healthcare Resource Utilization	Cases	Cases	Controls	Unadjusted Model		Adjusted Model	
				IRR (95% CI)		IRR (95% CI)	
**VHL-CNS-Hb (220 cases, 1,100 controls)**							
**Number of visits**							
Inpatient admissions	18.7	1.1	0.2	5.3 (3.8, 7.5)	***	4.7 (3.3, 6.7)	***
Inpatient days	175.2	9.3	0.9	10.0 (5.9, 16.9)	***	11.5 (6.3, 21.2)	***
Outpatient visits	64.3	17.8	8.9	2.0 (1.8, 2.2)	***	1.6 (1.4, 1.8)	***
ED visits	4.8	1.7	0.7	2.4 (1.6, 3.6)	***	2.0 (1.3, 3.0)	**
Other medical visits^1^	7.2	3.2	1.2	2.8 (1.8, 4.1)	***	2.7 (1.8, 4.2)	***
**VHL-pNET (20 cases, 100 controls)**							
**Number of visits**							
Inpatient admissions	37.4	2.1	0.2	10.2 (4.4, 23.7)	***	10.8 (5.3, 22.1)	***
Inpatient days	355.5	15.4	0.4	39.5 (13.2, 117.8)	***	29.6 (8.2, 106.4)	***
Outpatient visits	203.5	24.4	7.9	3.1 (2.5, 3.9)	***	1.8 (1.2, 2.7)	**
ED visits	2.3	0.9	0.9	1.0 (0.4, 2.6)		0.9 (0.3, 2.6)	
Other medical visits^1^	2.3	2.0	0.7	3.0 (1.6, 5.6)	***	2.7 (1.3, 5.6)	**

**Abbreviations**: CI: confidence interval; CNS: central nervous system; ED: emergency department; Hb: hemangioblastoma; HRU: healthcare resource utilization; IRR: incidence rate ratio; pNET: pancreatic neuroendocrine tumor; VHL: von Hippel-Lindau.

**Notes**:

* denotes P-values < 0.05; ** denotes P-values < 0.01; *** denotes P-values < 0.001

1. “Other” medical visits include all those not captured by inpatient, ED, or outpatient visits. These claims may reflect home health aids or physician visits among other types of ancillary medical claims

2. A claim was defined as “tumor-related” if it also had a corresponding diagnosis associated with it. See Jonasch et al. for the list of codes used for each tumor type [7]

3. The reported incidence rates for tumor-related HRU represent the estimated rates of each HRU category in 100 person-years due to the variable nature of patients’ follow-up lengths after index date

**Table 3:** Healthcare Costs for Patients with VHL-CNS-Hb or VHL-pNET

	Tumor-Related Healthcare Costs^3^	All-Cause Predicted Costs		All-Cause Cost Difference			
Annual Healthcare Costs (2020 USD)	Cases	Cases	Controls	Unadjusted Model		Adjusted Model	
**VHL-CNS-Hb (220 cases, 1,100 controls)**							
**Total costs** ^**1**^	-	$70,924	$11,418	$59,505	***	$49,645	***
**Medical costs**	$22,379	$61,919	$9,094	$52,825	***	$45,090	***
Inpatient costs	$19,145	$39,548	$3,915	$35,633	***	$36,430	***
Outpatient costs	$2,437	$16,750	$4,210	$12,540	***	$9,115	***
ED costs	$300	$3,657	$730	$2,927	***	$2,309	***
Other costs^2^	$497	$1,963	$239	$1,724	***	$1,689	***
**Pharmacy costs** ^**1**^	-	$9,005	$2,325	$6,681	***	$4,206	***
**VHL-pNET (20 cases, 100 controls)**							
**Total costs** ^**1**^	-	$97,661	$8,782	$88,879	***	$56,580	*
**Medical costs**	$22,325	$81,359	$5,906	$75,453	***	$46,538	*
Inpatient costs	$16,003	$50,087	$1,258	$48,829	***	$34,585	*
Outpatient costs	$6,286	$28,298	$3,951	$24,347	***	$16,731	*
ED costs	$30	$2,357	$583	$1,773	**	$2,026	**
Other costs^2^	$6	$617	$114	$503	**	$879	**
**Pharmacy costs**	-	$16,302	$2,876	$13,426	**	$8,703	*

**Abbreviations**: CNS: central nervous system; ED: emergency department; Hb: hemangioblastoma; pNET: pancreatic neuroendocrine tumor; VHL: von Hippel-Lindau; USD: United States dollars

**Notes**:

* denotes P-values < 0.05; ** denotes P-values < 0.01; *** denotes P-values < 0.001

1. Because pharmacy claims do not have corresponding diagnosis codes, the tumor-related total costs and pharmacy costs could not be calculated

2. “Other” medical costs include all those not captured by inpatient, ED, or outpatient costs. These claims may reflect home health aids or physician visits among other types of ancillary medical claims

3. A claim was defined as “tumor-related” if it also had a corresponding diagnosis associated with it. See Jonasch et al. for the list of codes used for each tumor type [7]

During the study period, the VHL-pNET cohort incurred a higher mean number of inpatient admissions (2.1 vs. 0.2; adjusted IRR [95% CI] = 10.8 [5.3, 22.1]; *P* < 0.001), outpatient visits (24.4 vs. 7.9; adjusted IRR [95% CI] = 1.8 [1.2, 2.7]; *P* < 0.01), and other medical visits (2.0 vs. 0.7; adjusted IRR [95% CI] = 2.7 [1.3, 5.6]; *P* < 0.01) per person-year, as well as longer mean inpatient length of stay (15.4 vs. 0.4 days per person-year; adjusted IRR [95% CI] = 29.6 [8.2, 106.4]; *P* < 0.001), relative to the control cohort (Table 2). Accordingly, higher total annual healthcare costs were observed in the VHL-pNET cohort compared to the control cohort ($97,661 vs. $8,782; adjusted cost difference = $56,580; *P* < 0.05), with medical costs representing the largest component ($81,359 vs. $5,906; adjusted cost difference = $46,538; *P* < 0.05; Table 3).

Patients in the VHL-CNS-Hb cohort had 18.7 inpatient admissions (including 175.2 inpatient days), 64.3 outpatient visits, 4.8 ED visits, and 7.2 other visits related to VHL-CNS-Hb per 100 person-years, resulting in mean annual medical costs of $22,379 (Tables 2 and 3).

Patients in the VHL-pNET cohort had 37.4 inpatient admissions (including 355.5 inpatient days), 203.5 outpatient visits, 2.3 ED visits, and 2.3 other visits related to VHL-pNET per 100 person-years, resulting in mean annual medical costs of $22,325 (Tables 2 and 3).

### Healthcare costs for surgery

Among all patients in the VHL-CNS-Hb cohort, even those who did not receive a particular surgery, mean annual costs for the surgical removal of VHL tumors ranged from $59 (laser therapy to the retina) to $9,297 (surgical removal of CNS-Hb), with average hospitalization costs among those patients who did receive surgery being highest for surgical removal of CNS-Hb ($94,439) and surgical removal of pNET tumors ($56,451; Table 4).

**Table 4:** Surgery Costs of Patients with VHL-CNS-Hb or VHL-pNET

	Unadjusted Costs for VHL-CNS-Hb Cohort		Unadjusted Costs for VHL-pNET Cohort	
Healthcare Costs (2020 USD)	(*N* = 220)		(*N* = 20)	
	Mean (SD)	Median [IQR]	Mean (SD)	Median [IQR]
**Nephrectomies**				
All patients (mean cost, annualized)	$408 (± $2,038)	$0 [$0, $0]	--	--
Average hospitalization cost for a nephrectomy procedure	$36,249 (± $10,568)	$35,443 [$29,338, $42,256]	--	--
**Laser therapy to retina**				
All patients (mean cost, annualized)	$59 (± $441)	$0 [$0, $0]	$10 (± $43)	$0 [$0, $0]
Average outpatient cost for laser therapy to retina	$2,234 (± $2,899)	$1,154 [$738, $1,691]	$699 (± $0)	$699 [$699, $699]
**Surgical removal of CNS-Hb**				
All patients (mean cost, annualized)	$9,297 (± $38,566)	$0 [$0, $0]	--	--
Average hospitalization cost for surgical removal of cerebellar and spinal Hb	$94,439 (± $70,100)	$81,786 [$54,598, $92,065]	--	--
**Surgical removal of adrenal pheochromocytomas**				
All patients (mean cost, annualized)	$123 (± $1,060)	$0 [$0, $0]	$9,837 (± $25,607)	$0 [$0, $0]
Average hospitalization cost for surgical removal of adrenal pheochromocytoma	$39,291 (± $367)	$39,399 [$38,882, $39,592]	$87,880 (± $42,471)	$83,659 [$53,115, $122,646]
**Surgical removal of pNET tumors**				
All patients (mean cost, annualized)	$221 (± $2,327)	$0 [$0, $0]	$16,917 (± $24,222)	$899 [$0, $33,846]
Average hospitalization cost for surgical removal of pNET tumor	$56,451 (± $23,288)	$56,451 [$39,984, $72,918]	$62,300 (± $39,900)	$59,634 [$26,113, $81,290]

**Abbreviations**: CNS: central nervous system; Hb: hemangioblastoma; IQR: interquartile range; pNET: pancreatic neuroendocrine tumor; SD: standard deviation; USD: United States dollar; VHL: von Hippel-Lindau.

Among all patients in the VHL-pNET cohort, mean annual costs for the surgical removal of VHL tumors ranged from $10 (laser therapy to the retina) to $16,917 (surgical removal of pNET tumors), with average hospitalization costs among those patients who did receive surgery being highest for surgical removal of adrenal pheochromocytomas ($87,880) and surgical removal of pNET tumors ($62,300; Table 4). There were no nephrectomies or surgical removals of CNS-Hb observed among patients with VHL-pNET.

### Costs associated with tumor reduction procedure complications

**Fig. 2:**
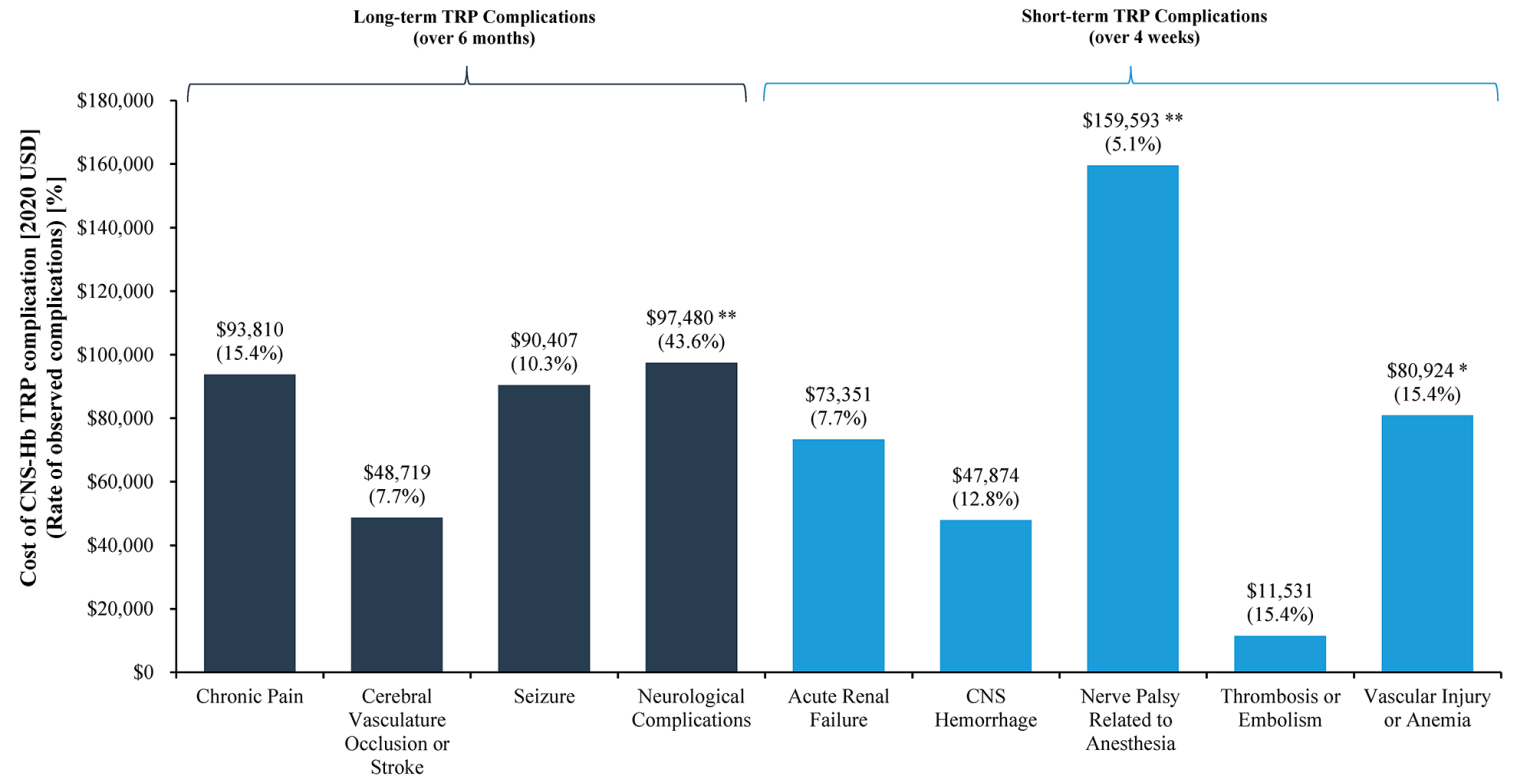
Rates and Costs Associated with CNS-Hb Tumor Reduction Procedure Complications^1,2,3^. Abbreviations: CNS: central nervous system; Hb: hemangioblastoma; PNET: pancreatic neuroendocrine tumor; TRP: tumor reduction procedure; USD: United States dollar; VHL: von Hippel-Lindau. Notes: * denotes P-values < 0.05; ** denotes P-values < 0.01. (1) In total, 39 tumor reduction procedures were considered in this analysis. Proportions are calculated with the total number of tumor reduction procedures as the denominator. (2) Complications associated with CNS-Hb reduction procedures were classified as any associated diagnoses or procedures related to a medical complication following a CNS-Hb reduction procedure. (3) Costs of tumor reduction procedure complications were estimated via univariate generalized linear models, using a Tweedie distribution and log link function. These models compared costs between patients who had a complication versus those who did not

Among patients who underwent CNS-Hb tumor reduction procedures in the cohorts of patients with VHL-CNS-Hb, the costliest long-term complications over 6 months were neurological complications ($97,480; observed in 43.6% of tumor reduction procedures), chronic pain ($93,810; observed in 15.4% of procedures), and seizure ($90,407; observed in 10.3% of procedures; Fig. 2). The costliest short-term tumor reduction procedure complication over 4 weeks was nerve palsy related to anesthesia ($159,593; observed in 5.1% of tumor reduction procedures).

Since the number of complications from tumor reduction procedures observed in the pNET cohort was very small, costs were not evaluated. The most common long-term complication observed among VHL-pNET patients was exocrine pancreatic insufficiency or secondary diabetes (20%), while the most common short-term complications were postoperative infections (20%) and respiratory complications (40%).

## Discussion

In this retrospective, real-world study, prevalence was estimated to be 1.12 cases per 100,000 for VHL-CNS-Hb and 0.12 cases per 100,000 for VHL-pNET based on a claims-based algorithm. Additionally, patients with VHL-CNS-Hb or VHL-pNET were found to incur substantial HRU compared with control patients, resulting in $49,645 and $56,580 higher total annual healthcare costs, respectively. Notably, costs related to VHL-CNS-Hb or VHL-pNET accounted for an important proportion of the total costs, and hospitalizations for the surgical removal of CNS-Hb and pNETs were particularly expensive. Lastly, complications resulting from CNS-Hb tumor reduction procedures further added to the considerable disease and economic burden of VHL disease.

The claims-based algorithm identifying patients with VHL disease was used in a previous study, in which the prevalence of VHL-associated RCC (VHL-RCC) was estimated to be 0.92 patients per 100,000 [7]. This prevalence is lower than that of VHL-CNS-Hb (1.12 patients per 100,000) but higher than that of VHL-pNET (0.12 patients per 100,000) estimated in the current study, which is generally consistent with the proportion of VHL patients that develops each type of tumor (CNS-Hb: 70-80%, RCC: 25-70%; pNET: 9-17%) [1, 8]. Additionally, based on the results from the previous study that assessed the economic burden of patients with VHL-RCC, HRU was generally similar between patients with VHL-RCC and those with VHL-CNS-Hb, while patients with VHL-pNET appeared to have a higher incremental incidence of inpatient admissions (adjusted IRR of 10.8) relative to patients with VHL-RCC (adjusted IRR of 4.7), though the magnitude of the results for patients with VHL-pNET may have been driven by the small cohort. Moreover, the incremental number of inpatient days appeared to be higher for both patients with VHL-CNS-Hb (11.5) and VHL-pNET (29.6) in the present study compared to those with VHL-RCC (5.1). Accordingly, VHL-CNS-Hb and VHL-pNET were both associated with considerable annual healthcare costs ($49,645 and $56,580, respectively) relative to VHL-RCC ($36,450) [7]. 

Together with the previous study, the present analysis represents the first to assess the prevalence and economic burden of patients with VHL-associated tumors within commercial insurance plans in the United States. To our knowledge, there are no prior estimates of prevalence for CNS-Hb or pNET (whether associated with VHL or not) in the existing literature, though incidence rates have been previously reported [14, 15]. However, these epidemiological estimates do not distinguish between VHL-associated and sporadic tumors so their comparability to the current study’s epidemiological findings are limited.

Similar to the epidemiology literature, there is limited research evaluating the economic burden of VHL-associated tumors, particularly CNS-Hb (whether related to VHL or not). Meanwhile, HRU and costs associated with NETs have been assessed in a few studies [16–18], though not specifically related to VHL-pNET. Chuang et al. evaluated cohorts of NET patients receiving either medical therapy or surgery and found that total monthly healthcare costs increased by 62% after diagnosis (from $5,630 to $9,093) for those treated with medical therapy and by 2.5 times (from $2,548 to $8,810) for those treated with surgery [16]. Moreover, in a chart review study of patients with NET, surgery was more common for those with pNET than those with other NET types (i.e., gastrointestinal and lung) [18]. Given the considerable costs found to be associated with VHL-related surgeries in the present study, these findings suggest that patients with VHL-pNET may have a particularly large economic burden relative to the general NET population. However, further study is warranted to generate additional estimates of the economic burden associated with VHL-associated tumors that can be compared to the present analysis.

### Limitations

Some limitations related to the VHL disease identification algorithm used in this study should be noted. Given the lack of an ICD VHL disease diagnosis code, the algorithm was validated based on physician notes and claims data, which may not include complete medical information, especially among patients with gaps in coverage and limited claims history. The limited nature of claims data in the United States means that the prevalence of VHL disease in this study and prior claims-based studies [7] is likely an underestimate of VHL’s true prevalence, which may in turn have affected the sample sizes of VHL patients in this study. The rarity of VHL disease and subsequently small validation sample of patients with a confirmed diagnosis from EHR further limited the algorithm validation process. As such, the algorithm was finalized based on both the EHR cohort validation and clinical input from medical experts regarding the management of VHL disease.

While regression models were adjusted for baseline covariates in the comparisons between the VHL-CNS-Hb/VHL-pNET and control cohorts, confounding may have occurred from unmeasured or unavailable covariates. Reasons for observing or not observing outcomes were not available in the claims data. Additionally, since the study was conducted among patients with commercial insurance, the results may not be generalizable to those with Medicare, Medicaid, or no insurance coverage. Lastly, although VHL-associated tumors likely have an important burden on overall daily function and employment, these outcomes were not evaluated in the current study.

## Conclusion

In this retrospective, claims-based study, the prevalence of VHL-CNS-Hb was estimated to be 1.12 cases per 100,000, while the prevalence of VHL-pNET was estimated to be 0.12 cases per 100,000 in the US in 2019. Both VHL-CNS-Hb and VHL-pNET were associated with substantial HRU and healthcare costs. These findings provide important insight for healthcare payers regarding the expected real-world costs that enrollees with VHL-CNS-Hb and VHL-pNET may incur over the course of their disease.

### Electronic supplementary material

Below is the link to the electronic supplementary material.

Supplementary Material 1

## Data Availability

The datasets generated and analyzed during the current study are not publicly available because they were used pursuant to a data use agreement. The data are available through requests made directly to Optum.
