# Supplementary material for: Epidemiology and economic burden of Von Hippel-Lindau Disease-associated central nervous system hemangioblastomas and pancreatic neuroendocrine tumors in the United States
Source: Orphanet J Rare Dis. 2024 Feb 16;19:73. doi: 10.1186/s13023-024-03060-w (PMC10873931; doi:10.1186/s13023-024-03060-w)
Supplement: Supplementary file 1 — Supplementary Material 1 [file 13023_2024_3060_MOESM1_ESM.docx]

# SupplementaRY Materials

Supplementary Figure S1. National Prevalence of VHL-CNS-Hb and VHL-pNET in 2019

**Abbreviations**: CNS: central nervous system; Hb: hemangioblastoma; pNET: pancreatic neuroendocrine tumor; VHL: von Hippel-Lindau.

**Note**:

1. The proportion of the US population in each age-gender stratum was sourced from the following reference: US Census Bureau, Current Population Survey, Annual Social and Economic Supplement, 2019.
